# Unbiased, scalable sampling of protein loop conformations from probabilistic priors

**DOI:** 10.1186/1472-6807-13-S1-S9

**Published:** 2013-11-08

**Authors:** Yajia Zhang, Kris Hauser

**Affiliations:** 1 School of Informatics and Computing, Indiana University, Bloomington, Indiana, USA

**Keywords:** Conformation sampling, Monte Carlo methods, protein loops, ensemble generation, graphical models

## Abstract

**Background:**

Protein loops are flexible structures that are intimately tied to function, but understanding loop motion and generating loop conformation ensembles remain significant computational challenges. Discrete search techniques scale poorly to large loops, optimization and molecular dynamics techniques are prone to local minima, and inverse kinematics techniques can only incorporate structural preferences in adhoc fashion. This paper presents Sub-Loop Inverse Kinematics Monte Carlo (SLIKMC), a new Markov chain Monte Carlo algorithm for generating conformations of closed loops according to experimentally available, heterogeneous structural preferences.

**Results:**

Our simulation experiments demonstrate that the method computes high-scoring conformations of large loops (*>*10 residues) orders of magnitude faster than standard Monte Carlo and discrete search techniques. Two new developments contribute to the scalability of the new method. First, structural preferences are specified via a probabilistic graphical model (PGM) that links conformation variables, spatial variables (e.g., atom positions), constraints and prior information in a unified framework. The method uses a sparse PGM that exploits locality of interactions between atoms and residues. Second, a novel method for sampling sub-loops is developed to generate statistically unbiased samples of probability densities restricted by loop-closure constraints.

**Conclusion:**

Numerical experiments confirm that SLIKMC generates conformation ensembles that are statistically consistent with specified structural preferences. Protein conformations with 100+ residues are sampled on standard PC hardware in seconds. Application to proteins involved in ion-binding demonstrate its potential as a tool for loop ensemble generation and missing structure completion.

## Background

Sampling conformations of kinematic chains -- rigid objects connected by articulated joints -- is a fundamental problem in protein structure prediction, the geometry of folding linkages, and robot motion planning. Sampling poses a challenging computational problem when chains are large and must satisfy a variety of constraints and statistical preferences. Conformations may be required to satisfy hard feasibility constraints, such as loop closure and collision avoidance, while also obeying soft preference constraints, such as low energy and high structural likelihood. Particularly around folded protein structures, the subset of feasible and favorable conformations comprises a miniscule fraction of the conformation space, and due to the "curse of dimensionality" this fraction shrinks dramatically with the dimensionality of the state space. Because interesting biological macromolecules have large numbers of degrees of freedom, ranging up to hundreds or thousands, new techniques are needed to sample severely constrained conformations efficiently.

Protein loops are flexible structures that often deform during binding, and are extremely important for understanding protein functioning [1]. Loop sampling has been used in missing fragment reconstruction, generating fluctuations in equilibrium conformations, and generating decoy sets for function prediction. Such applications typically require methods for sampling energetically-likely and diverse configuration *ensembles *rather than optimizing a single point estimate. The loop closure constraint, which requires the terminal atoms of a loop to lie at fixed positions dictated by the surrounding structured regions, poses a major challenge in sampling. Existing loop sampling methods include discrete search [2], optimization [1], and inverse kinematics (IK) methods [3-6]. Fiser et al's [1] approach takes an energy function that encodes spatial restraints and preferences on dihedral angles, and then runs a numerical optimization to minimize that energy. Optimization is relatively computationally expensive, which is usually worthwhile for single structure prediction but less so for generating conformation ensembles. Discrete search methods are able to explore a wider space of conformations by incrementally building a tree of clash-free subchain conformations starting from one end of the loop and progressing toward the terminal end [2,7,8]. Although effective for small loops, these methods do not scale well to large loops due to the problem of combinatorial explosion. As a result, discrete search is intractable with chains containing 7-8 or more residues. They also introduce discretization artifacts, and are not able to close the gap between a terminal atom and its desired position. Inverse kinematics (IK) techniques from the robotics field have been adopted to sample conformations with *exact *loop closure [3-5,9]. However, these methods prioritize the loop closure constraint and do not take energies into account during sampling. Hence, some authors employ a secondary energy optimization step to generate more plausible conformations [3,6,10].

For each of these methods, the sampling *distribution *is throughly entangled with the sampling *procedure*. To achieve a desired distribution, the sampling procedure must be tuned in an opaque, nontrivial manner, and it is unclear that a desired distribution can even be achieved. Instead, extensive post-hoc empirical testing to assess the quality of the resulting distribution and to argue that a method samples well. Monte Carlo (MC) techniques represent a more principled class of approaches that sample directly from a desired destribution. They have a long history of use in computational biology because they can quickly explore multiple energy minima and transition pathways, while molecular dynamics and optimization techniques often get stuck in single local minima [11-14]. They are also well-suited for generating conformation ensembles. The general Metropolis-Hastings approach generates a sequence of incrementally perturbed configurations via a random walk, with a carefully-designed acceptance criterion (the *detailed balance *condition) that ensures that the sampling distribution approaches the desired one as more samples are drawn. However, there is a tradeoff in choosing the perturbation size: small perturbations raise the fraction of accepted moves but lower the speed of conformation space exploration. Moreover, standard MC techniques cannot be directly applied to protein loops due to the loop closure constraint, which causes each step to be accepted with probability 0.

Our new method overcomes many of the weaknesses of prior methods (see Table 1); it simultaneously scales to long loops (e.g., *>*10 residues) and produces unbiased ensembles of conformations. It uses a unified probabilistic graphical modeling (PGMs) framework for modeling a desired distribution from experimentally available statistical priors. PGMs such as Bayesian networks and Markov random fields are powerful tools for inference in large domains with heterogeneous sources of information, and have been applied in a limited sense to protein structure prediction. They have been used to predict side-chain rotamer conformations [15] and conformations of macromolecular assemblies from electron density maps [16]. Their use is reasonably well understood in the discrete case, but continuous variables often prove challenging. Our work derives a new Gibbs sampling inference method for continuous PGMs with loop-closure constraints, which restrict the feasible domain to a nonlinear implicit manifold. In particular we derive the mathematical relationship between an inverse kinematic sampling distribution and the manifold's metric tensor, which is necessary to compute the detailed balance condition in the Metropolis-Hastings algorithm. The resulting sampling sequence is *unbiased *in the sense that its distribution approaches the target distribution in the large-sample limit.

**Table 1:** Characteristics of loop generation techniques

Technique	Loop closure	Prior distribution/energy function	Global search	Scalability
Optimization	Exact	Y	N	+
Inverse kinematics sampling	Exact	N	Y	++
Discrete search	Inexact	Y	Finite subset	-
Standard Monte Carlo	No	Y	Y, reqs. mixing	+
SLIKMC	Exact	Y	Y, reqs. mixing	++

## Methods

SLIKMC is a Markov chain Monte Carlo (MCMC) method that takes as input an experimental conformation scoring function Φ, a protein structure from the Protein Data Bank (PDB), the beginning and ending residues of the loop, and outputs a sequence of perturbed loop conformations such that the sequence asymptotically approaches a probability distribution proportional to Φ. If the structure is missing, a rough initial structure is sampled using existing inverse kinematics loop closure techniques. To generate a subsequent conformation, it performs the following operations:

For each 4-residue subloop, repeat the following steps:

1. Sample a new subloop conformation that satisfies kinematic constraints.

2. Compute the Metropolis-Hastings importance ratio *α *of the new conformation against the previous conformation.

3. Accept or reject the new subloop conformation with probability *α*.

The method terminates when a fixed number of conformations are generated or until a desired time cutoff is reached. The novel contributions of this paper include an exact derivation of the importance ratio *α *for the inverse kinematics sampler of step 1 and the use of sparse PGMs to evaluate the importance ratio quickly per-subloop. We also describe extensions that handle flexibility in side-chains and molecules with multiple branches or loops (e.g., polycyclic compounds).

As a MCMC method, SLIKMC samples from a complex joint probability distribution by constructing a Markov chain whose equilibrium distribution is equal to the desired distribution. It is a hybrid MCMC algorithm that combines blocked Gibbs sampling and Metropolis-Hastings (M-H) sampling. M-H permits the use of non-normalized probability distributions, which is important because it is relatively simple to define a useful scoring function but virtually impossible to ensure that it integrates to one. The blocked Gibbs sampling method samples a small subloop at each step, which helps SLIKMC scale better to large chains, because acceptance rates decrease roughly exponentially in the number of variables sampled at once. This section will first review classical MCMC methods and then describe the new approach.

### Markov chain Monte Carlo framework

Let **x **= (*x*_1_, ..., *x_n_*) denote the state variables of the system of interest. Experimental conditions including hard constraints and soft preferences are encoded into a non-negative scoring function Φ(*x*_1_, ..., *x_n_*). The score is required to have finite integral, is zero at states that violate hard constraints, and higher values indicate more desirable states. Φ is considered as an unnormalized probability density, and our goal is to generate samples with probability proportional to Φ. In other words, the goal is to sample from the normalized density *P *defined as:

(1) $$P\left(x_{1},...,x_{n}\right)=\frac{1}{Z}\Phi \left(x_{1},...,x_{n}\right)$$

where the *proportionality constant Z *that ensures that *P *integrates to 1. Φ is closely related to energy functions *E*(**x**) through the Gibbs measure

(2) Φ(x)=exp(-E(x)/T)

where *T *is the system temperature.

The Metropolis-Hastings (M-H) algorithm addresses the problem that it is hard to sample directly from an unnormalized distribution Φ in part due to the difficulty of evaluating the normalization term *Z *[17]. On step *k*, M-H first samples a candidate move from **x**^(*k*) ^to **x**′ from a *proposal distribution Q*(**x**′; **x**^(*k*)^), and *accepts *the move **x**^(*k*+1) ^*← ***x**′ with probability

(3) $$\alpha =\text{min}\left(1,\frac{P\left(x^{\prime }\right)Q\left(x^{\left(k\right)};x^{\prime }\right)}{P\left(x^{\left(k\right)}\right)Q\left(x^{\prime };x^{\left(k\right)}\right)}\right).$$

This is the so-called *detailed balance *condition. The *Z *terms in the numerator and denominator cancel out, so we use Φ directly instead of *P*. If the move is rejected, then the current state is maintained: **x**^(*k*+1) ^*← ***x**^(*k*)^. The term

(4) $$\frac{\Phi \left(x^{\prime }\right)Q\left(x^{\left(k\right)};x^{\prime }\right)}{\Phi \left(x^{\left(k\right)}\right)Q\left(x^{\prime };x^{\left(k\right)}\right)}$$

is called the *importance ratio*. If the ratio is greater than 1, then the new sample is accepted; otherwise it is accepted with probability equal to the ratio. With the detailed balance construction, *P *(**x**) is indeed the stationary distribution of the Markov chain generated by successive samples.

The key question for M-H is how to choose a proposal distribution that we can sample from and evaluate. The acceptance strategy must evaluate the terms in (3) exactly so that the M-H algorithm respects the detailed balance. One of our key contributions is a technique for evaluating *Q *exactly when sampling from closed chain submanifolds, which enables our method to generate an unbiased sampling sequence.

Note that it is challenging to choose *Q *to approximate *P *closely, and hence in practice the probability of accepting a sample drops sharply in the dimensionality of the space. Gibbs sampling is commonly used to address this issue. Moreover it is convenient when sampling from a conditional density is easier than sampling from the entire joint density, which is the case in the sparse Bayesian models that we employ in this work. Given the current sample $x^{\left(k\right)}=\left(x_{1}^{\left(k\right)},...,x_{n}^{\left(k\right)}\right)$ at time *k*, Gibbs sampling generates the next state **x**^(*k*+1) ^by sampling a single variable $x_{i}^{\left(k+1\right)}$ from the conditional density

(5) $$P\left(x_{i}^{\left(k+1\right)}|x_{1}^{\left(k+1\right)},...,x_{i-1}^{\left(k+1\right)},x_{i+1}^{\left(k\right)},...,x_{n}^{\left(k\right)}\right)$$

and keeping the remaining variables fixed. The variable is updated and the index *i *is incremented in looping fashion. If the dependencies between the variables are sparse (e.g., every variable *x_i _*only depends on a handful of variables rather than the remaining *n *- 1 variables), then Gibbs sampling can be efficient even for very large problems. This is exploited in sparse PGMs. Blocked Gibbs sampling is a variation of Gibbs sampling that groups multiple variables as a block and samples the block from the joint distribution conditioned on all other variables.

Our method combines Gibbs sampling with M-H sampling to generate a new sample from (5). To do so, simply consider all other variables fixed, sample $x_{i}^{\prime }$ from a conditional proposal distribution $Q\left(x_{i}^{\prime };x_{i}|x_{1},...,x_{i-1},x_{i+1},...,x_{n}\right)$, and then apply the importance ratio test as usual to determine whether to accept the step $x_{i}^{\left(k+1\right)}\leftarrow x_{i}^{\prime }$ or keep $x_{i}^{\left(k+1\right)}=x_{i}^{\left(k\right)}$.

### Sparse factored models

Due to the locality of interactions in most scoring functions of interest, it is possible to represent Φ in a *factored *form:

(6) $$\Phi \left(x_{1},...,x_{n}\right)=\underset{i}{\prod }\phi_{i}\left(S_{i}\right)$$

where each *ϕ_i _*is known as a *factor *and each *S_i _*is a subset of {*x*_1_, ..., *x_n_*} known as the *domain *of the factor *ϕ_i_*. For example, in protein structure prediction factors may include Ramachandran plots relating each pair of dihedral angles (*φ*, *ψ*), steric clashes, energy functions defined over atom positions, and prior knowledge from B-factors or electron density maps.

Probabilistic graphical models like Bayesian networks and Markov random fields are inherently factored: the domain of each factor consists only of a vertex and its neighbors in the graph. A graphical model is *sparse *if each variable *x_i _*is involved in only a handful of factors (i.e., bounded by a constant unrelated to *n*), and hence only interacts directly with a few other variables. An important consequence in the discrete case is that probabilistic inference is computationally tractable in sparse models (polynomial in *n*), whereas inference is intractable in dense models (in general, exponential in *n*). A key step in our method converts the representation of a kinematic chain from dense to sparse form, as described below. The implementation described in this paper currently supports:

• Ramachandran plots *ϕ*_*RP*(*r*)_(*φ*, *ψ*) which vary by residue *r*.

• Steric clashes *ϕ*_*SC*(*j*, *k*)_(*p_j_*, *p_k_*) which are 0 if atom *j *collides with atom *k *and 1 otherwise.

• B-factors defined as Gaussians $\phi_{BF(j)}(p_{j})=\frac{1}{c\sqrt{2\pi B_{j}}}\exp-\left(\frac{||p_{j}-\mu_{j}|{|}^{2}}{2B_{j}c^{2}}\right)$ where *µ_j _*is the predicted atom position and *B_j _*is the B-factor value in the protein's PDB file. A constant of proportionality *c *can be set by the user according to his/her confidence in the quality of the B-factor estimates.

• Side-chain rotamer distributions, as described the Side Chain Sampling section.

Each factor can be evaluated quickly, but over thousands or millions of evaluations they accumulate significant computational cost. Significant savings can be achieved in sparse models, because when a few variables are changed, the change in Φ can be calculated quickly by only evaluating those factors involved, rather than recomputing Φ from scratch. Although steric clashes are theoretically considered as *O*(*n*^2^) pairwise factors, in practice we use a grid-based hashing data structure that only checks nearby atoms for collision. As a result, each Gibbs sampling step can be performed in *O*(1) time.

In future work we are interested in including additional statistical potentials and/or all-atom energy function terms in scoring. With a naive implementation, each atom is involved in *O*(*n*) pairwise interactions, but we expect to exploit the weakness of distant interactions to reduce the number of factors included in the computation.

### Kinematic chain modeling

Consider a jointed kinematic chain with reference frames *T*_0_, *T*_1_, ..., *T_N_*, connected with relative rotational angles *q*_1_, ..., *q_N_*. For a protein backbone, there is a one-to-one correspondence between frames and backbone atom positions *p*_1_, ..., *p_M_*, and the rotational variables are simply the backbone dihedral angles *φ*_1_, *ψ*_1_, ..., *φ*_*N*/2_, *ψ*_*N*/2_.

Although it is standard practice and beneficial for certain algorithms to define the system state with a minimal set of coordinates, e.g., **x **= (*T*_0_, *q*_1_, ..., *q_N_*), a key step of our method is to consider an *expanded state*. Minimal coordinates use the fact that each subsequent frame *T*_1_, ..., *T_N _*can be determined from **x **through straightforward forward kinematics, leading to a lower dimensional representation. However, this approach eliminates sparsity in the probabilistic model because a factor defined on *T_N _*will depend on all variables, a factor defined over *T*_*N - *1 _will depend on all variables except *q_n_*, and so on. Moreover, if a sampler is asked to generate certain variables from a density defined over *T*_1_, ..., *T_N _*(for example, atom positions), the generated distribution may be biased unless it computes the determinant of an *N × N *metric tensor for each evaluation of Φ. As described below, this is a consequence of nonlinear transformations of distributions (see Appendix). On the other hand, computing determinants takes *O*(*N*^3^) time, which scales poorly with large *N*.

**Figure 1:**
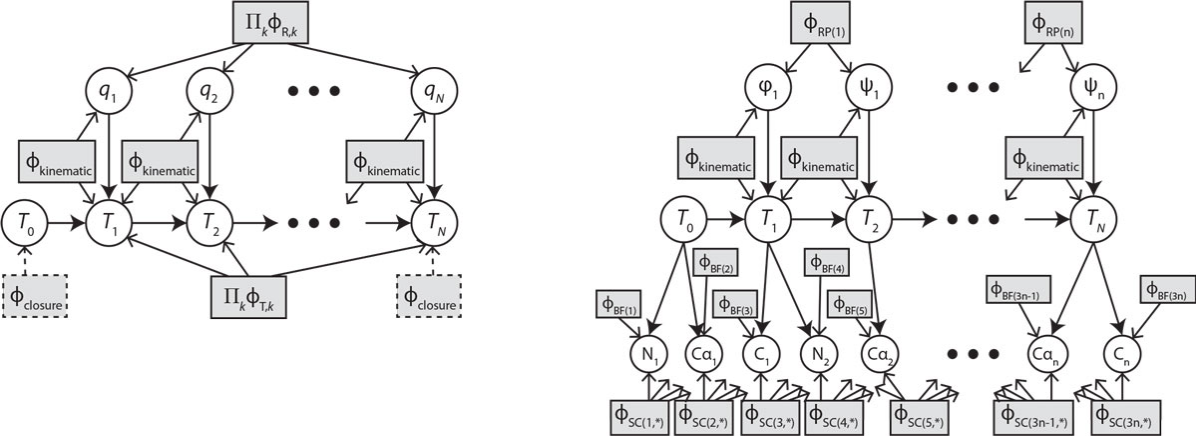
**Probabilistic graphical models of kinematic chains**. Left: sparse graphical model relating *N *joint angles and link transformations via local factors. Right: instantiation of the model for an *n*-residue protein backbone, with an additional layer accounting for atom positions.

Our method represents an expanded state that incorporates all spatial variables along with the conformation variables: **x **= (*q*_1_, ..., *q_N_*, *T*_0_, ..., *T_N_*). The joint probability density is then defined over angles and reference frames of all links along the chain (see Figure 1):

(7) $$\Phi \left(x\right)=\underset{i}{\prod }\phi_{i}\left(S_{i}\right)\overset{N}{\underset{j=1}{\prod }}\phi_{kinematic}\left(T_{j}|T_{j-1},q_{j}\right)$$

where each *S_i _*is now a subset of {*q*_1_, ..., *q_n_*, *T*_0_, ..., *T_n_*}, and where *ϕ_kinematic _*is the forward kinematic transform that defines the frame *T_j _*in terms of the prior frame *T*_*j*-1 _and the relative angle *q_j_*. Because the transform is deterministic, *ϕ_kinematic _*should be thought of as an indicator function. Taking the convention that each frame's origin lies on its joint's axis:

(8) $$\phi_{kinematic}\left(T_{j}|T_{j-1},q_{j}\right)=\left\{\begin{array}{cc} 1 & \text{if}\;T_{j}=T_{j-1}T_{j}^{rel}R\left(a_{j},q_{j}\right)\\ 0 & \text{otherwise} \end{array}\right)$$

where $T_{j}^{rel}$ is the relative transformation of frame *j *relative to frame *j - *1 and *R*(*a*, *q*) is the rotation of angle *q *about axis *a*. Fixed-endpoint constraints can also be encoded with indicator factors *ϕ_closure_*(*T*_0_) and *ϕ_closure_*(*T_n_*) that are zero everywhere except at the fixed frames.

With (7) encoded so that factors contain few variables in their domain, the model becomes sparse. However, we have added the complication of maintaining a valid kinematic structure, because the set of **x **for which Φ is nonzero lies on a lower-dimensional manifold. Technically speaking, the probability density must be considered with respect to a base measure that assigns finite, nonzero density to the manifold. For 3D chains, the state space has dimensionality 7*N *but the manifold has dimensionality 6 + *N *for free-endpoint chains or *N - *6 for fixed-endpoint chains. The next section will describe how we handle these submanifolds in detail.

### Block sampling and selection

A block is a subset of variables that are simultaneously sampled. The number of variables in a block must be sufficiently large to give at least one continuous degree of freedom of movement. The Metropolis-Hastings criterion is used to accept or reject a move because it is unrealistic to sample directly from the block's conditional density. This key subroutine, **Sample-Block-MH**, takes as input the previous sample **x**^(*k*) ^and a block *B *of *b *consecutive joint angles and their intervening frames. It then samples a candidate move, and accepts it according to the M-H criterion. Pseudocode is as follows:

**Sample-Block-MH**(**x**^(*k*)^, *B*):

1. Using **Sample-Block **as described below, sample a candidate conformation ${x^{\prime }}_{B}$ of *B *at random, keeping the rest of the chain $x_{C}^{\left(k\right)}$ fixed.

2. Compute the M-H acceptance probability

$$\alpha =\min\left(1,\frac{\Phi_{B}({x^{\prime }}_{B})Q_{B}(x_{B}^{\left(k\right)}|x_{C}^{\left(k\right)})}{\Phi_{B}(x_{B}^{\left(k\right)})Q_{B}({x^{\prime }}_{B}|x_{C}^{\left(k\right)})}\right).$$

3. Accept the move $x_{B}^{\left(k+1\right)}\leftarrow {x^{\prime }}_{B}$ with probability *α*.

Here the subscript *B *denotes the subset of variables in the block, while the subscript *C *denotes the complement of the block. The score Φ*_B _*calculates the product of factors *ϕ_i _*whose domains *S_i _*overlap with *B*, which is more efficient than recomputing Φ from scratch. The remaining details of the method -- the block size, the block sampling procedure, and calculating the sampling probability *Q_B _*-- are described in detail in the remainder of this section. To generate a new conformation **x**^(*k*+1) ^of the entire chain, **Sample-Block-MH **is called several times with overlapping blocks incremented sequentially down the chain. Block ordering (e.g. forward, backward, or random order) has no effect on the asymptotic distribution and experiments suggest virtually no noticeable effect apart from the first handful of samples. Thanks to sparsity, each pass is performed in *O*(*N*) time, which takes a fraction of a second for chains with hundreds of variables.

How many variables should be included in a block? Standard Gibbs sampling (i.e., *b *= 1) does not work because loop closure constraints constrain the conditional density of any variable given the rest (5) to a Dirac. Hence, the state would never change. In fact, no mixing occurs for *b ≤ *5, except possibly at singular conformations, which occupy a set of measure zero in conformation space and are therefore unlikely to occur naturally. For 6 angles, analytical inverse kinematics (IK) techniques are available to compute solutions for a pair of fixed end frames [9]. In fact, any number from 0 to 16 solutions may exist for a given 6-angle problem. Nevertheless, *b *= 6 is not suitable because it restricts the random walk to only a finite set of conformations.

**Figure 2:**
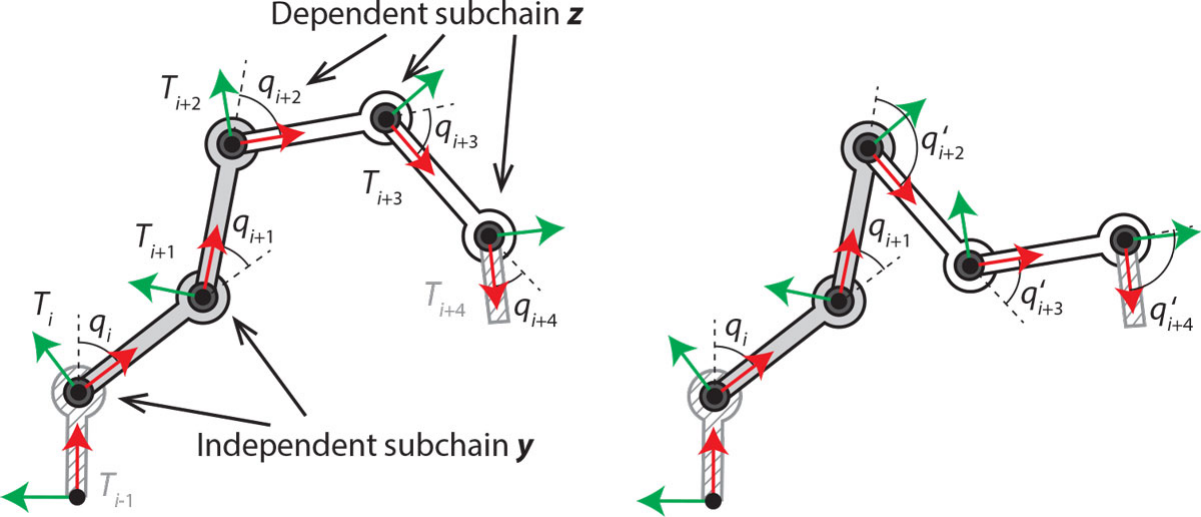
**Parameterization of subloops via independent subchains**. Left: a 5-angle block for a planar chain with fixed end frames *T*_*i*-1 _and *T*_*i*+4_. Right: a second IK solution for the dependent subchain.

Setting *b *= 7 angles allows sufficient freedom to sample from a 1-dimensional manifold of solutions. In general, a block of *b ≥ *6 angles admits a *b - *6 dimensional solution manifold. Denote the block *B *= {*q_i_*, ..., *q*_*i*+*b-*1_, *T_i_*, ..., *T*_*i*+*b-*2_}, and let us call the first *b - *6 angles of the block *q_i_*, ..., *q*_*i*+*b*-7 _the *independent *subchain. Call the remaining 6 angles the *dependent *subchain. This is illustrated for a planar chain in Figure 2. A sampling procedure is as follows:

### Sample-Block

1. Sample values for the independent subchain at random.

2. Attempt to close the chain by calculating an analytical IK solution for the dependent subchain. We use the method of [9].

3. If more than one IK solution exists, one is picked at random, and if no solution exists, the process terminates with failure.

**Figure 3:**
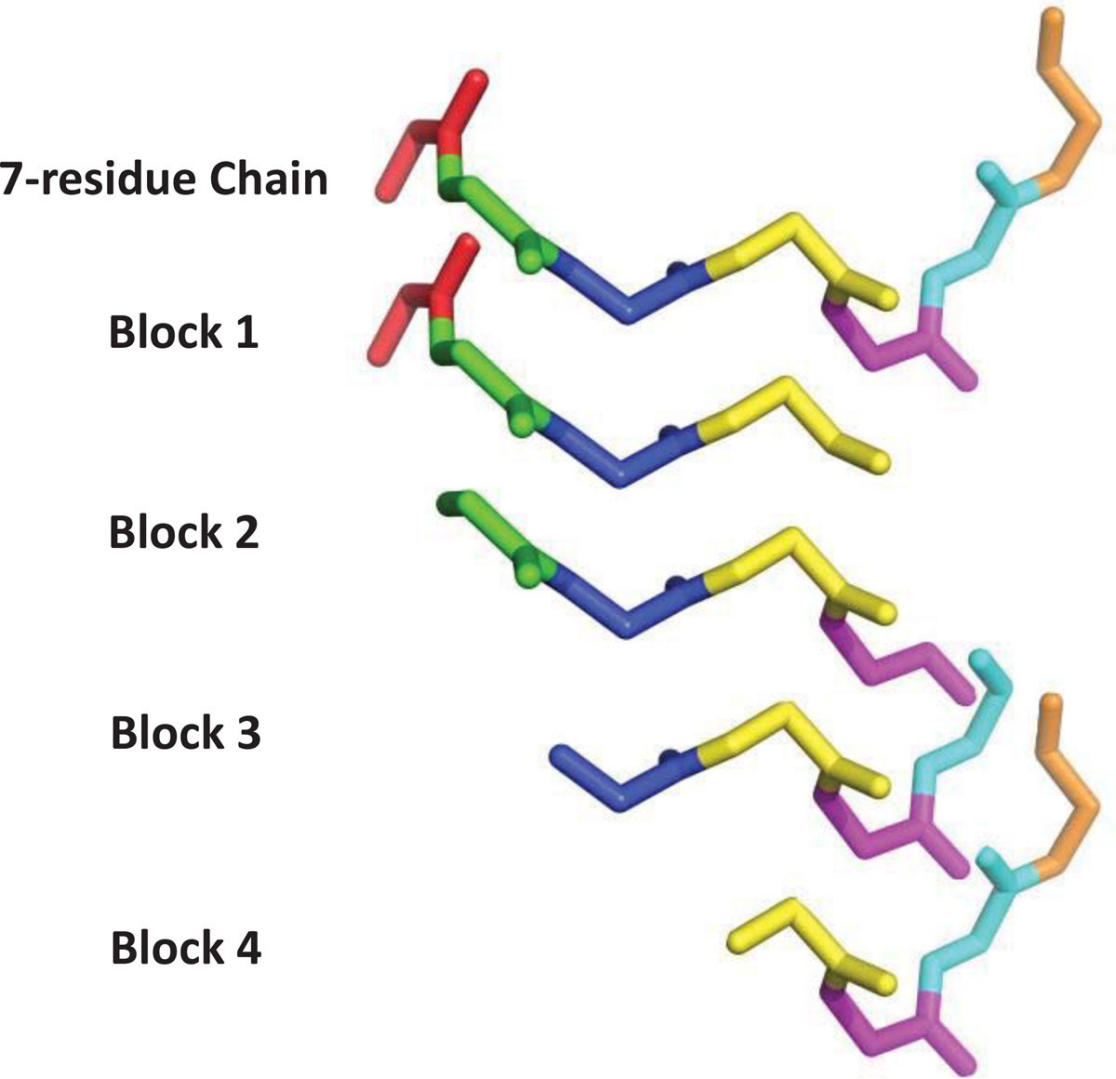
**Block selection**. A 7-residue chain is shown with each residue drawn in a distinct color. SLIKMC incrementally samples block of 4 consecutive residues (8 torsional angles) with the first 3 residues overlapping with the preceding block.

It is recommended that *b ≥ *7 be chosen as low as possible, because as *b *grows, the probability of sampling an independent subchain that admits closure drops off dramatically as *b *grows, particularly for "stretched out" conformations. In our implementation, 4 consecutive residues are considered as a block that contains *b *= 8 angles since even numbers align better with the (*φ*, *ψ*) angles priors of each residue (see Figure 3). (Throughout this discussion we have assumed a 3D chain but the method works equally well in 2D. For planar chains, at least 4 angles are needed, and the manifold of solutions is (*b - *3)-dimensional)

### Calculation of sub-loop sampling densities

To calculate the M-H importance ratio, we must calculate *sampling density *for the *sampling procedure ***Sample-Block**. Several concepts from differential geometry are required in order to derive this density $Q_{B}\left({x^{\prime }}_{B}|x_{C}^{\left(k\right)}\right)$.

**Figure 4:**
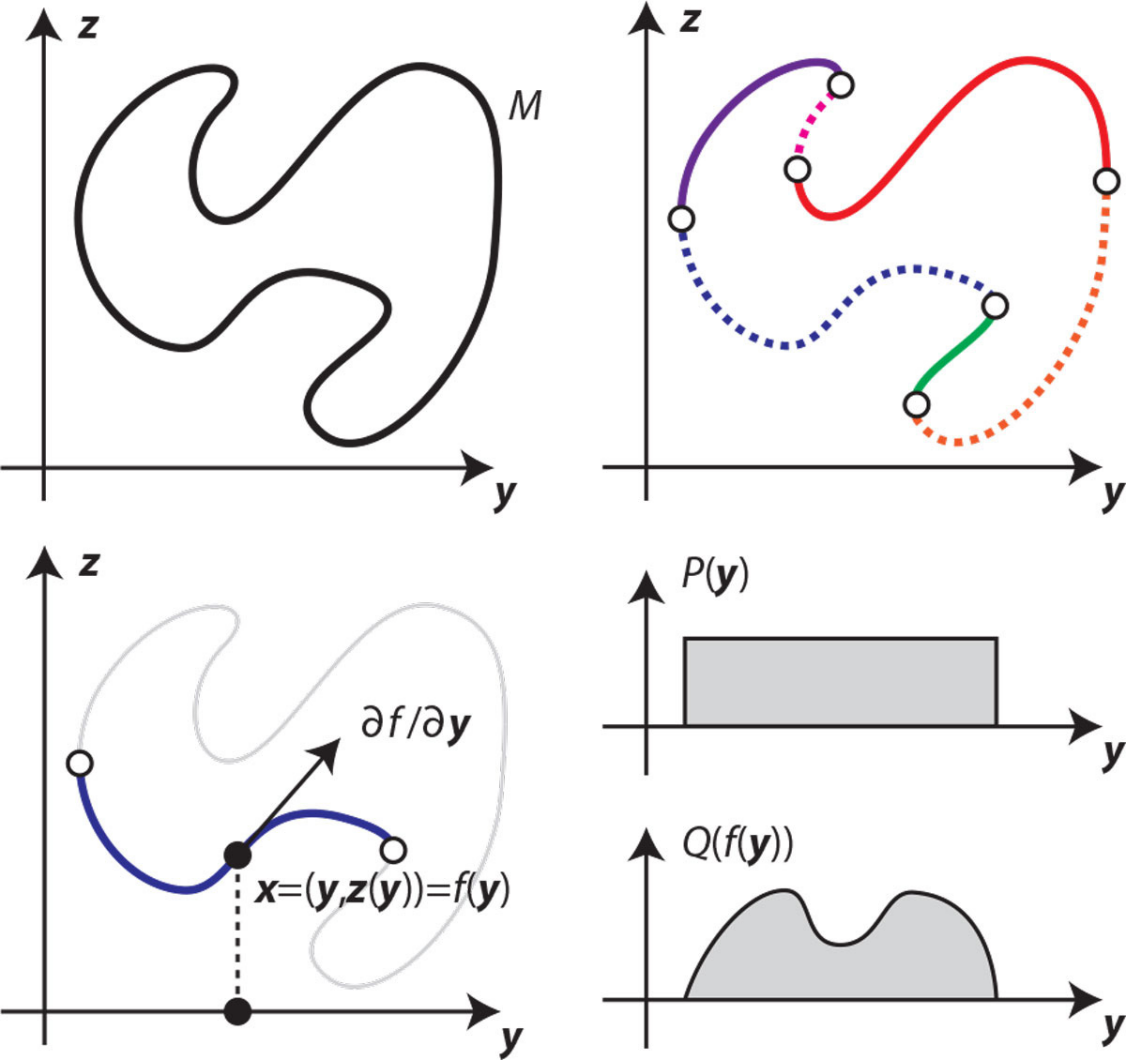
**Sampling distributions on manifold charts**. Top: abstract illustration of how analytical IK implicitly decomposes a 1-parameter manifold *M *into a set of local bijections (charts). Bottom: the Jacobian of a chart must be taken into account when calculating the sampling distribution *Q *over *M*.

Fix the endpoints of the block, and let *M *denote the (*b - *6)-dimensional manifold of loop-closing conformations. Let us call the (*b - *6) angles of the independent subchain **y**, which are sampled w.r.t. the density *P *(**y**). Observe that the candidate sample ${x^{\prime }}_{B}$ is distributed according to a nonlinear transformation of *P*(**y**) onto *M*. In fact, at non-singular conformations the independent subchain forms a local *chart *of *M*, which is a local bijection between ${\mathbb{R}}^{b-6}$ to *M *centered at ${x^{\prime }}_{B}$ (see Figure 4). Since there is a local bijection *f *between **y **and the point on the manifold **x***_B_*, the sampling density over **x***_B _*is given by:

(9) $$Q_{B}\left(x_{B}|x_{C}^{\left(k\right)}\right)=\frac{P\left(y\right)}{s\sqrt{\text{det}G\left(y\right)}}$$

where *s *is the number of IK solutions at **y **and *G *is the *metric tensor *of the chart **x***_B _*= *f*(**y**) (see Appendix). The inclusion of the metric tensor is a natural consequence of transformation of variables. For example, for the case *b *= 7, the metric tensor is the squared arc length of the 1-dimensional parametrization of *M *(Figure 4, bottom). In general, *G *is given by

(10) $$G\left(y\right)={\left(\frac{\partial f}{\partial y}\left(y\right)\right)}^{T}W\left(\frac{\partial f}{\partial y}\left(y\right)\right)$$

where $\frac{\partial f}{\partial y}\left(y\right)$ is the Jacobian of the function *f*. Here we have also introduced a positive semidefinite weighting matrix *W *for the purpose of weighting the relative importance of matching the prior along certain axes. In the standard case, *W *is an identity matrix, but it can also be useful to choose a nonuniform diagonal matrix to account for heterogeneous units (e.g., angle vs. position variables).

A remaining issue is that it is often difficult to explicitly compute the Jacobian of the IK function involved in *f*. In other words, with **z ***≡ ***z**(**y**) denoting the 6 angles in the dependent chain, it is difficult to evaluate *∂***z***/∂***y**. So, we compute an *implicit chart Jacobian *by considering the implicit form of the constraints *C*(**x***_B_*) = 0. These vector-valued constraints state that the difference between the terminal frame of the subchain and the desired frame is zero.

We have the constraint equation:

(11) $$0=C\left(x_{B}\right)=C\left(y,z\right)$$

Taking the derivative of both sides of (11) with respect to **y **we get:

(12) $$0=\frac{\partial C}{\partial y}+\frac{\partial C}{\partial z}\frac{\partial z}{\partial y}$$

and hence

(13) $$\frac{\partial z}{\partial y}=-{\left(\frac{\partial C}{\partial z}\left(x_{B}\right)\right)}^{-1}\frac{\partial C}{\partial y}\left(x_{B}\right)$$

holds as long as $\frac{\partial C}{\partial z}$ is invertible, which is true everywhere except at singular conformations. Each derivative of *C *in the above expression is a submatrix of the Jacobian and can be computed using standard techniques.

Finally, since

(14) $$f{\left(y\right)}^{T}=\left[y^{T},z^{T},T_{i},...\;T_{i+b-2}\right]$$

we obtain the Jacobian

(15) $$\frac{\partial f^{T}}{\partial y}=\left[I,\frac{\partial z^{T}}{\partial y},\frac{dT_{i}}{dy},...,\frac{dT_{i+b-2}}{dy}\right]$$

in which *I *is the identity matrix and all frame derivatives are calculated using the chain rule $\frac{dT_{j}}{dy}=\frac{\partial T_{j}}{\partial y}+\frac{\partial T_{j}}{\partial z}\frac{\partial z}{\partial y}$. These partial derivatives are calculated using standard techniques.

Beyond computing the proper sampling density, it is also important to design the algorithm to efficiently compute the M-H acceptance probability. Since clash detection takes 60 times more computation time than calculating the rest of the terms in Φ, we check collisions *after *determining whether a move will be accepted. Compared to the naive method, this method achieves an order of magnitude speedup.

### Extension to other topologies

Although the core method applies to linear closed kinematic chains, it can be extended to handle other molecular topologies, such as free-endpoint chains and side-chains. In theory, polycyclic compounds may also be handled as well. Each new topological structure requires specialized block selection and sampling routines. For example, free-end-point chains need separate sampling subroutines for the start and end blocks. Standard MC methods are used to do so.

Side-chain deformations are important for shaping binding cavities, and SLIKMC can be adapted to generate side-chain conformations in the same graphical modeling framework. It is known that the side-chain conformation depends on the backbone dihedral angle of the corresponding residue [18]. This requires sampling side-chains after the backbone conformation is sampled. Furthermore, since the distribution of side-chain torsional angles are limited to small number of typical conformations (rotamers) for most residues [19], we sample side-chains according to experimentally-determined distributions.

#### Side-chain sampling

For side-chain conformation priors we use the 2010 Backbone-dependent Rotamer Library [20]. In this library, each rotameric residue is associated with a list of rotamers which representing the high probability regions for side-chain torsion angles. The probability of a rotamer conformation *χ *is modeled as a continuous distribution given the backbone dihedral angle pairs. The dihedral angle (*φ*, *ψ*) space of each rotameric backbone residue *r *is discretized into a grid and each cell [*a*, *b*] *× *[*c*, *d*] contains its experimentally observed probabilities *P *(*χ | r*, *a ≤ φ ≤ b*, *c ≤ ψ ≤ d*). Each distribution over *χ *is specified as a Gaussian mixture model. For non-rotameric residues the terminal *χ *angles are handled specially due to the asymmetry in their distributions.

Treating the remainder of the protein as fixed, we model the target distribution of a side-chain **x***_s_*, conditional on backbone dihedral angles **x***_b_*, as follows:

(16) $$\Phi_{s}\left(x_{s}|x_{b}\right)\;=\phi_{SC}\left(x_{s}|x_{b}\right)\phi_{R\left(r\right)}\left(x_{s}|x_{b}\right)$$

where *ϕ_SC _*indicates steric-clashes and *ϕ*_*R*(*r*) _indicates the side-chain conformation prior for residue *r*. Side-chain B-factors are typically not included since we want to give enough freedom to explore the conformation space, and our experiments indicate that the flexibility of the protein chain will reduce greatly when we specify B-factors as prior to both backbone and side-chain atoms.

Extending block sampling to include side-chains requires justifying the importance ratio carefully to ensure unbiased sampling. An efficient sampling procedure is as follows: first compute a closed-loop backbone subchain from the blocked Gibbs sampling step and compute its acceptance probability as usual. If accepted, sample each side chain along the block according to its backbone-dependent rotameric distribution. Because it is a Gaussian mixture, we can sample from *ϕ*_*R*(*r*) _directly: pick a Gaussian from the mixture according to its weight and then sample from the Gaussian. Finally, reject the sample if the side chains collide.

To justify this procedure, we show that its acceptance probability is equal to the M-H acceptance probability for the entire block including side-chains. Let the block be **x***_B _*= (**x***_b_*, **x***_s_*) and a candidate block sample ${x^{\prime }}_{B}=\left({x^{\prime }}_{b},{x^{\prime }}_{s}\right)$, with *b*, *s *denoting backbone and side-chain variables respectively. The M-H importance ratio is

(17) $$I\left(x_{b},x_{s}\right)=\frac{\Phi \left({x^{\prime }}_{b},{x^{\prime }}_{s}\right)Q\left(x_{b}|x_{s}\right)}{\Phi \left(x_{b}|x_{s}\right)Q\left({x^{\prime }}_{b},{x^{\prime }}_{s}\right)}=\frac{\Phi_{b}\left({x^{\prime }}_{b}\right)\Phi_{s}\left({x^{\prime }}_{s}|{x^{\prime }}_{b}\right)Q\left(x_{b}\right)Q\left(x_{s}|x_{b}\right)}{\Phi_{b}\left(x_{b}\right)\Phi_{s}\left(x_{s}|x_{b}\right)Q\left({x^{\prime }}_{b}\right)Q\left({x^{\prime }}_{s}|{x^{\prime }}_{b}\right)}$$

by conditioning on **x***_b_*. Since we sample the side-chain according to *Q*(**x***_s_|***x***_b_*) = *ϕ*_*R*(*r*)_(**x***_s _| ***x***_b_*) and the prior sample is clash-free, we cancel terms in the numerator and denominator to get:

(18) $$I\left(x_{b},x_{s}\right)=\frac{\Phi_{b}\left({x^{\prime }}_{b}\right)Q\left(x_{b}\right)}{\Phi_{b}\left(x_{b}\right)Q\left({x^{\prime }}_{b}\right)}\phi_{SC}\left({x^{\prime }}_{s}|{x^{\prime }}_{b}\right)$$

Since the first term is simply the importance ratio of the backbone and *ϕ_SC _*is binary, we conclude that the block acceptance probability is either the backbone importance ratio if clash-free or zero if clashing. Hence the side-chain sampling procedure is sound.

#### Multiply-closed kinematic loops

It may be possible to extend SLIKMC to handle multiply-closed loops such as those that occur in polycyclic compounds. This requires special care to divide the structure into blocks that can be split into dependent and independent subchains, such that a conformation of the independent subset completely determines the dependent subset, up to some finite multiplicity. In other words, the independent subchains form a chart of the space of closed-chain conformations of the whole block. The union of all blocks must also cover all state variables.

**Figure 5:**
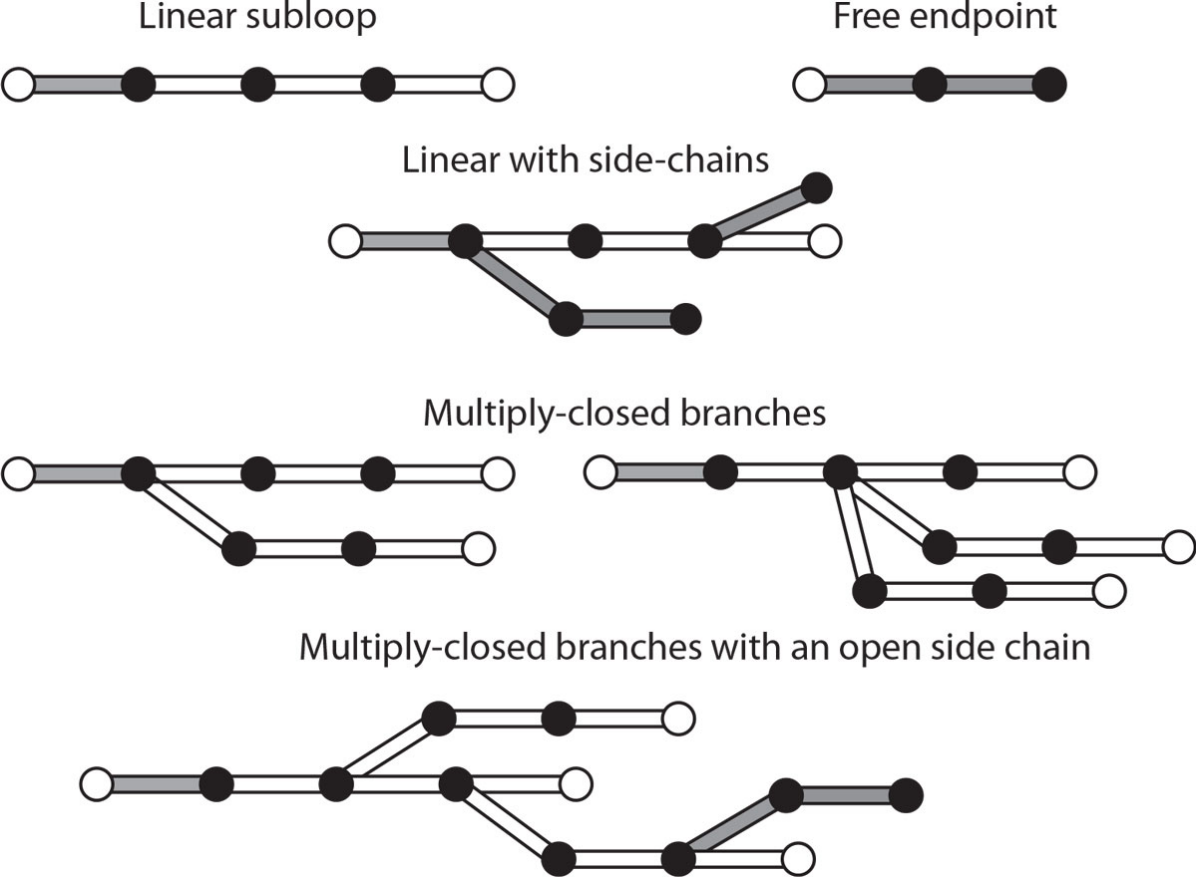
**Extending to non-linear topologies via branching blocks**. Several branching structures may be treated as blocks. Independent chains (shaded) must be chosen to parameterize the manifold of configurations satisfying closed chain constraints (open circles).

We illustrate the principle on planar kinematic chains, which require blocks of size at least 4. Assume each cycle contains at least 3 joints. We define a topological ordering by selecting a linear main chain and considering branches off of the main chain. Non-branching linear blocks, free-endpoint blocks, and side-chains (open-ended branches) are handled as described above. Each 3-joint branch off of a branching block is then considered as part of a dependent subchain (see Figure 5). Branches off the dependent subchain are also added to the block in a recursive manner, leading to a block with a tree topology.

**Figure 6:**
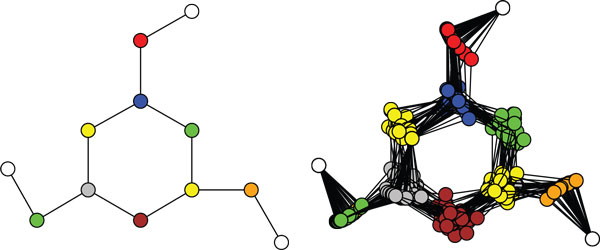
**Results on a planar multi-loop structure**. Fluctuations of a 2D chain with a closed ring constrained on the three ends (open circles). Left: initial conformation. The angular prior for each link is modeled as a normal distribution with 20° standard deviation. Right: 20 samples with skip length 100.

To sample a branching block, we first sample values for the independent subchain at random and then close the loops for each branch according to their topological order. To ensure unbiased sampling, we must also calculate the metric tensor in (9) for the entire branched block. This in turn requires computing the Jacobian of the chart, which requires computing the Jacobian of the implicit form for the multiple loop-closure constraints (11). Due to the tree structure the Jacobian is sparse, and the matrix inversion in the implicit chart Jacobian (13) can also be computed efficiently. We have implemented this approach on 2D chains with closed rings (see Figure 6), and extending it to 3D chains remains a problem for future work.

### Mixing and autocorrelation

In any MCMC method it is important to empirically examine the mixing rate of the Markov Chain. Firstly, it can potentially take many iterations to "forget" the effects of a poor initialization. For protein sampling, this is not a significant problem because we initialize the chain with the native structure in PDB, which is typically quite good.

**Figure 7:**
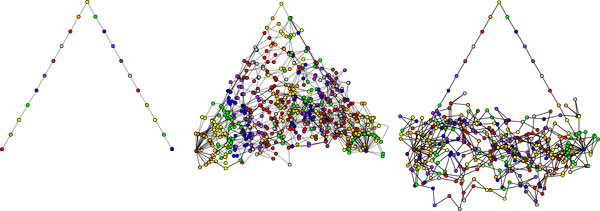
**Mixing of SLIKMC samples**. Sampling conformations of a planar 20-link chain, anchored at the endpoints, with a uniform prior. Left: starting from a deliberately bad initial conformation. Middle: the sequence mixes relatively quickly, but the first 40 samples are biased by the initial conformation and autocorrelate strongly. Right: a sequence that takes every 40'th sample does not significantly autocorrelate.

Secondly, subsequent samples are highly autocorrelated, and many conformations must be skipped to obtain a sequence with low autocorrelation. This is a serious concern because autocorrelation grows stronger as more variables are included in the conformation (see Figure 7). In practice, one must determine the skip length empirically in order to obtain a *quasi-independent *sampling sequence, which is defined as a sequence with autocorrelation below some given threshold (0.2 is used in our experiments).

## Result and discussion

**Figure 8:**
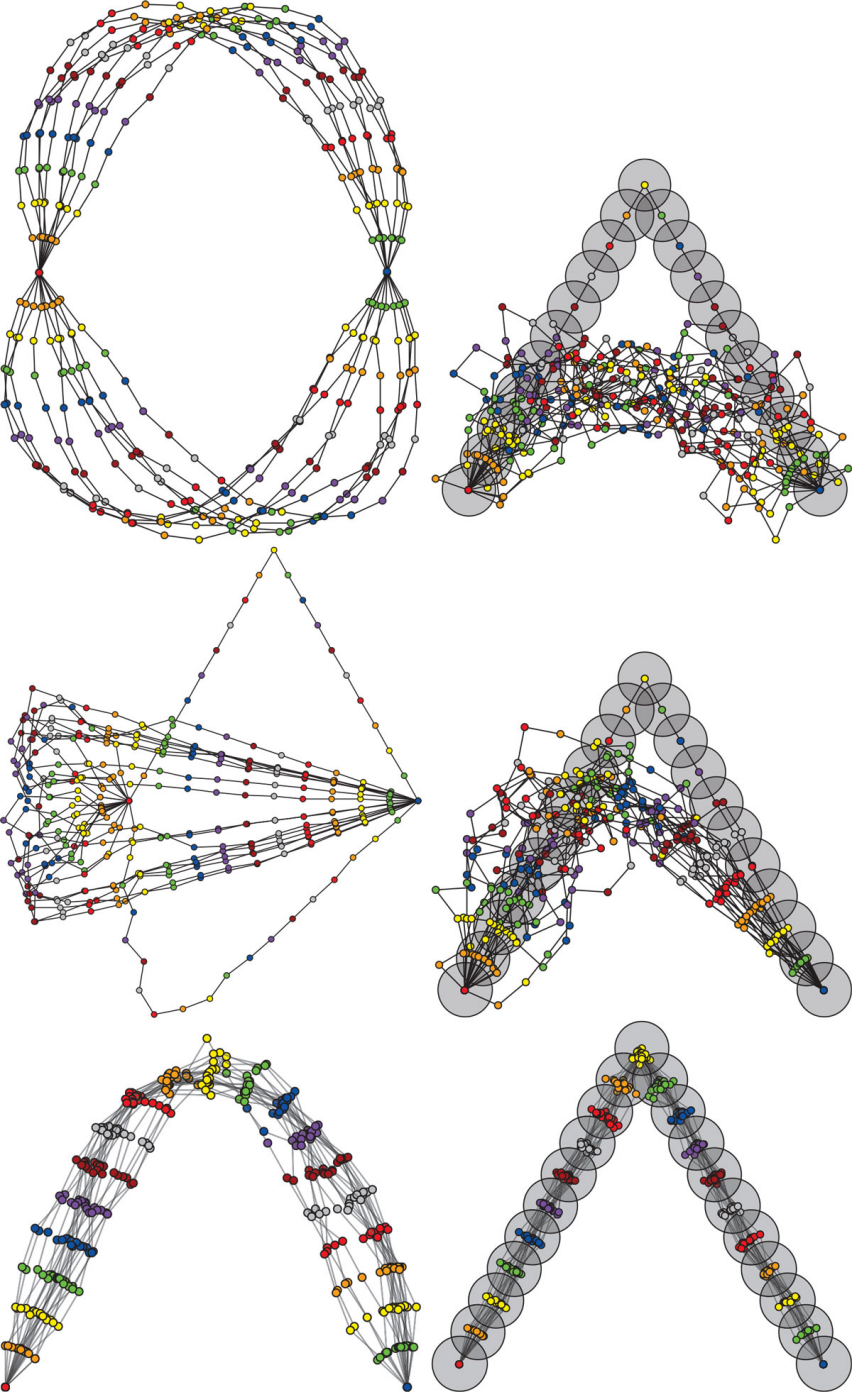
**Closed-chain sampling**. Three sampling methods for a 20-link closed-loop chain. At left, the prior gives preference to joint angles with small magnitude. At right, the prior gives preference to joint positions in a triangle shaped distribution (circle centers: means, shaded circles: 3*σ *spreads). Top: sampling joint angles followed by numerical loop closure, best 20/20,000 samples. Middle: sampling with RLG [5], best 20/20,000 samples. Bottom: SLIKMC, displayed every 40'th sample. These sample sets are generated by our method approximately as fast as RLG and an order of magnitude faster than numerical loop closure.

**Figure 9:**
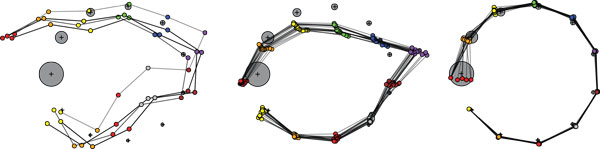
**Free-endpoint chain sampling with heterogeneous priors**. Comparing SLIKMC against a standard Metropolis-Hastings (M-H) sampler on a free-endpoint chain with heterogeneous prior distribution over joint positions (crosses: means, shaded circles: 3*σ *spreads). For each method, 400 iterations are run and every 20'th sample is retained, taking approximately 10 s time on a standard PC. Left: M-H takes steps that are too large and only generates 4 unique samples. Middle: M-H with step size reduced by 10 has a higher success rate but slower convergence. Note the lack of variance in the leftmost point. Right: our method.

The SLIKMC algorithm implements a scalable framework for Monte Carlo sampling of kinematic chains. The technique uses a blocked Gibbs sampler that proposes movements of small subchains of conformation angles at once, along with a Metropolis-Hastings technique that guarantees an unbiased sampling of the loop-closure submanifold for that block. Due to the small block size, each energy function is local and adjustments are fast, ranging from microseconds to milliseconds. The method is mathematically proven to generate a statistically unbiased sample in the large sample limit. It is particularly well-suited for closed loops (see Figure 8) but can also be advantageous for chains with free endpoints as well (see Figure 9).

SLIKMC is implemented as an add-on to the software package LoopTK [21][22] for protein loop sampling and is available at http://www.iu.edu/~motion/slikmc/. All experiments are run on a Intel i7 2.7 GHz computer with 4 GB RAM. The library currently supports sampling with prior information from Ramachandran plots, steric clashes, and B-factors, and supports integration with the Backbone-Dependent Rotamer Library for side-chain sampling. Numerical experiments suggest that SLIKMC generates higher quality samples for large loops with lower computational cost than standard Monte Carlo techniques for open-ended chains and the RAMP loop completion package [23].

### Loop sampling with prior distributions

**Figure 10:**
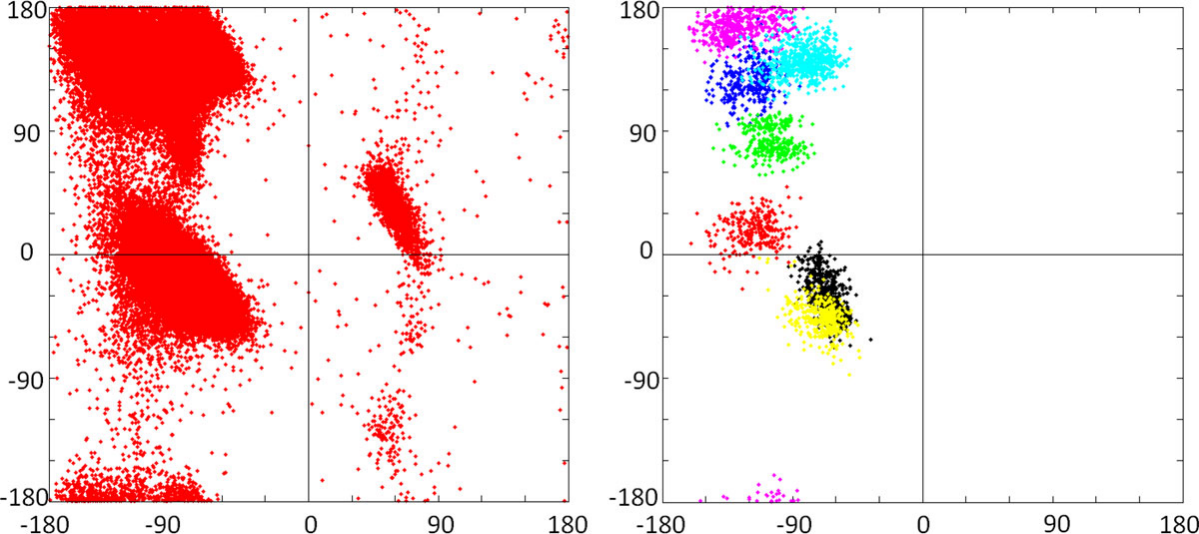
**Ramachandran plot of SLIKMC samples**. Left: the Ramachandran plot of generic residues from a database that includes 500 high-resolution proteins [26] used as a prior. Right: the Ramachandran plot for the generic residues in our 10-residue test protein (1AMP 181-190) generated from 2,000 consecutive samples. Each color represents one residue. (This figure is best viewed in color.)

We consider the 10-residue closed loop 1AMP181-190, which is a representative segment for testing loop reconstruction algorithms [24]. SLIKMC is applied to sample 2000 conformations from a joint probability that includes steric clashes, Ramachandran plots, and B-factors. The Ramachandran plot (see Figure 10) shows that the distribution of dihedral angles is contained within high probability regions but explores relatively widely, with an average angular deviation of 37°.

**Figure 11:**
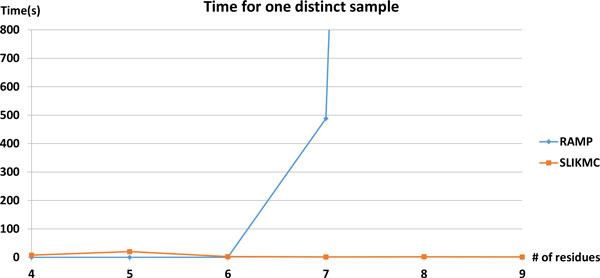
**Running time comparison between RAMP and SLIKMC**. Time required for the discrete search method RAMP and SLIKMC to obtain one sample for loops of varying size. The time required for RAMP increases exponentially while our method runs in approximately constant time.

We compared our method with the discrete-search loop construction software RAMP [8,23,25]. The latest version 0.7b was used in these experiments. We test the methods on loops of 1AMP with different lengths starting from residue 181. RAMP is tested by calling loop closure function with 0.5 Å distance tolerance. SLIKMC samples from perturbed segments using Ramachandran plots as prior with clash-free constraint. Figure 11 plots the average time for both methods to obtain one closed conformation. Due to combinational explosion, the time required for RAMP increases exponentially and is impractical for *>*6 residues.

**Figure 12:**
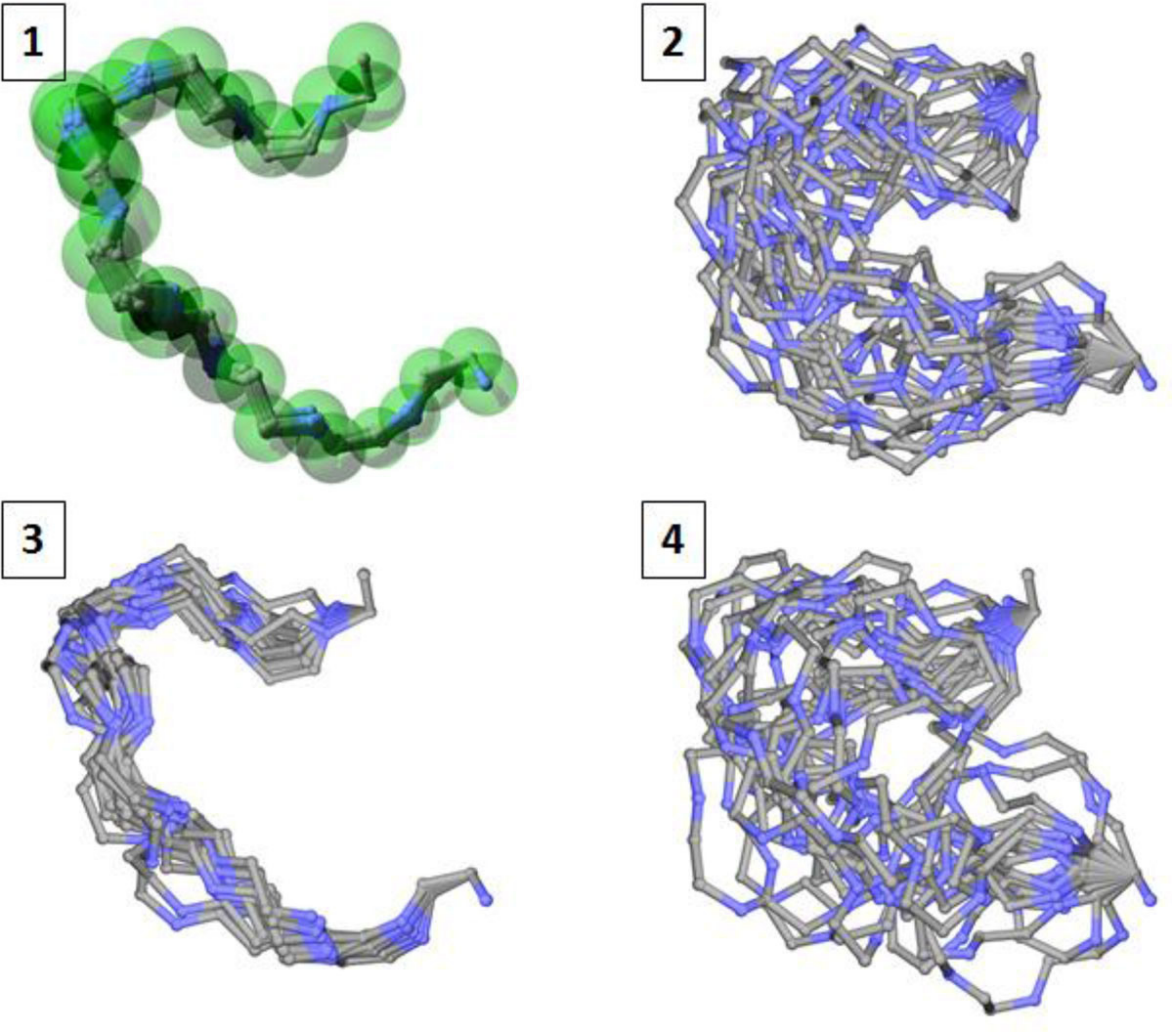
**Comparing SLIKMC against sample-then-select**. Left: samples generated by SLIKMC with a skip length of 100. Right: samples generated by post-selecting the top 20 scoring samples generated from the LoopTK IK sampler. Transparent balls depict the 3*σ *spread of the atom position prior derived from its B-factor. The top row uses the original B-factors, while the bottom row enlarges B-factors by 10.

**Figure 13:**
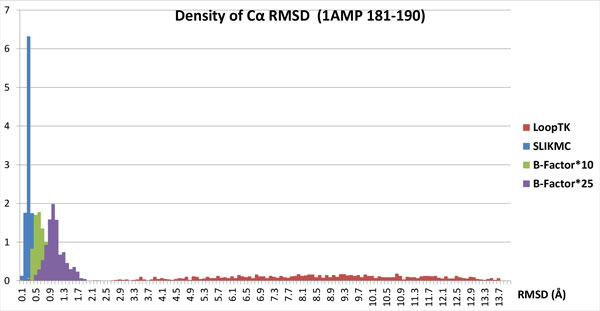
**RMSD distributions from SLIKMC against IK**. Histogram of RMSD to the native structure for samples from SLIKMC and the LoopTK sampler on 1AMP 181-190. With SLIKMC the use of prior information allows fine-grained control over the sampling distribution.

We also compare SLIKMC with a sample-then-select inverse kinematics method that first samples a set of clash-free, loop-closing conformations and then extracts the top scoring ones. The LoopTK configuration sampling method [22] was used here. Given 300 s cutoff time, LoopTK generates 888 conformations, while our method generates 705. Figure 12 shows how the top 20 samples of LoopTK compare with every 100th sample of our method. The upper figures are generated using the original B-factors in the PDB file. SLIKMC matches the prior information more closely and obviates the need for postprocessing using numerical optimization. The bottom figures correspond to enlarged B-factors, which indicate less confidence in the values prescribed by the PDB file. The distribution of SLIKMC samples has approximately three times the variance of the original, which closely matches theoretical predictions. Figure 13 shows the distribution of RMSDs of *C_α _*atoms compared to the native structure for both LoopTK and SLIKMC with varying scales in B-factors. This suggests that SLIKMC better supports variations in the experimenter's relative confidence in heterogeneous sources of information.

### Missing loop completion

**Figure 14:**
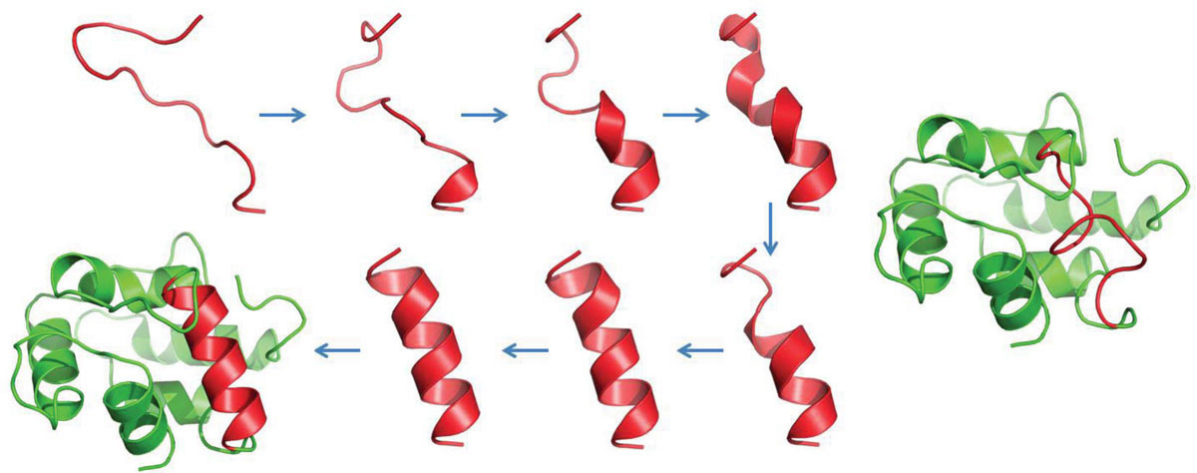
**Helix recovery**. Left: Starting from a highly perturbed conformation, SLIKMC recovers a helix using only clash and Ramachandran plots information. Every 20 samples are drawn. The final displayed conformation has RMSD 0.2704 to the PDB structure. Right: by comparison, an IK technique attains a minimum RMSD of 4.0655 out of 13,000 samples (90 minutes running time).

We now consider an application to completion of missing loops. Given the starting position and ending position of a missing segment, we first generate an arbitrary loop-closing configuration, then run SLIKMC to perturb it to a high-probability conformation. As a test case, we select a helix structure (residue from 40-51) from an APO protein 1B8C. We generate an arbitrary loop-closing configuration by running the LoopTK configuration sampling method [22] to perturb the original conformation. Starting from the highly disordered conformation, SLIKMC is run for 2 minutes using priors including enlarged B-factors (scaled by a factor of 10) from original chain segment and Ramachandran plots with steric clash-free constraints. SLIKMC generates approximately 300 samples within the time limit, and the closest sample to the original structure is with RMSD 0.2704 calculated from backbone atoms (see Figure 14). In contrast, the LoopTK configuration sampling method did poorly in constructing a favorable missing loop.

### Scalability tests on free-endpoint chains

**Figure 15:**
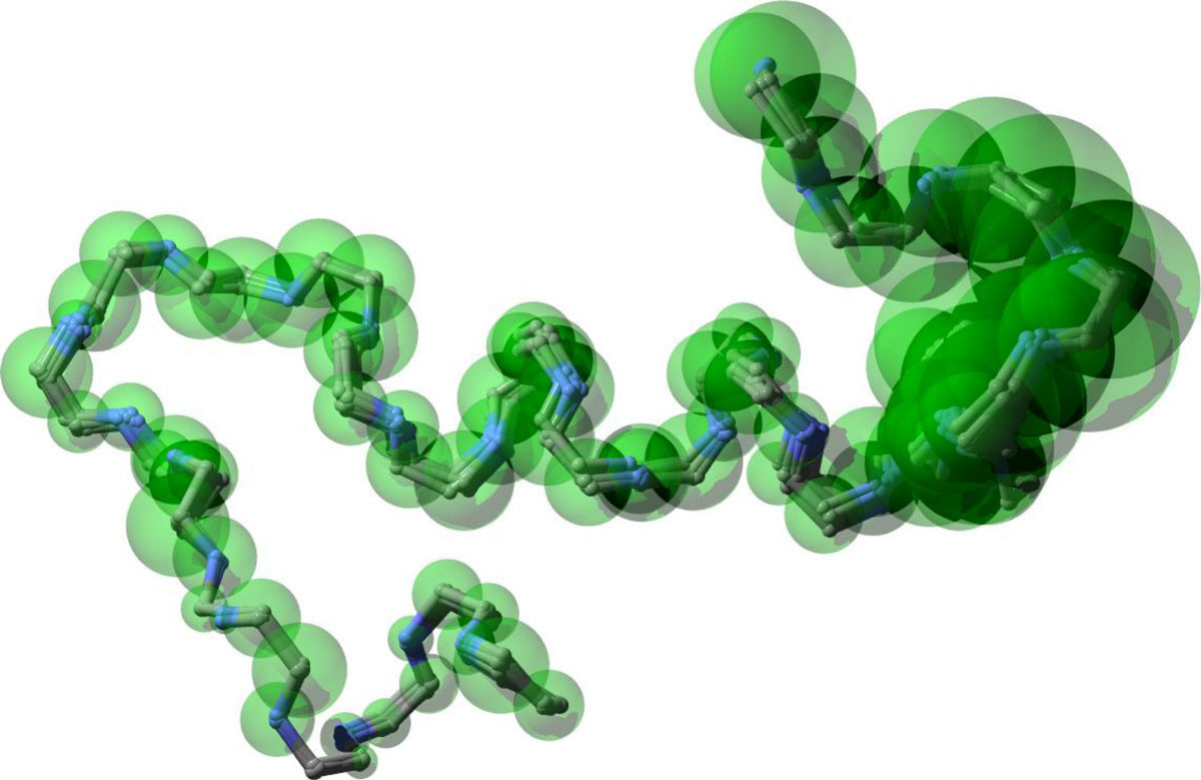
**Samples of a 30-residue chain**. 12 samples of a 30-residue subchain of protein 1B8C selected from the first 300 consecutive samples with skip length 25. Transparent balls depict the 3*σ *spread of the atom position prior derived from its B-factor. Atoms with low B-factors near the end of the chain increase the difficulty for a standard MC method to explore the conformation space.

**Figure 16:**
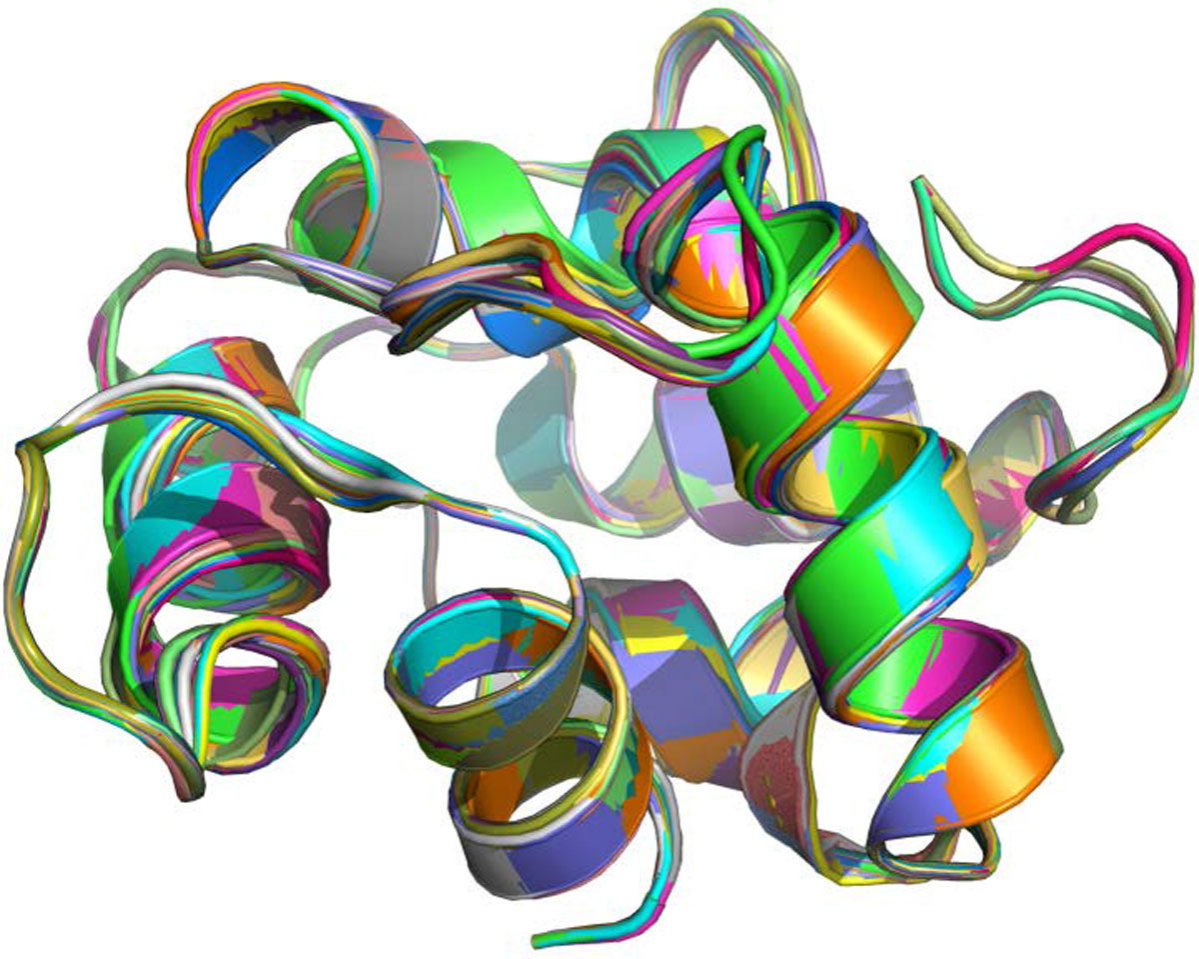
**Samples of a 108-residue chain**. 17 samples of 1B8C chain A (108 residues) selected from 170 consecutive samples with skip length 10. Each conformation is drawn in a distinct color.

To further study scalability, we apply SLIKMC to subchains of chain A in a calcium-binding protein 1B8C. Samples for a 30-residue subchain are generated in 1 s (Figure 15) and samples for the entire 108-residue 1B8C protein are generated in approximately 4 s (Figure 16).

We compare SLIKMC against a standard Metropolis-Hastings algorithm that samples backbone angles according to a Gaussian proposal distribution with 1° standard deviation. The target distribution for both methods includes steric clashes, Ramachandran plots, and B-factors. Note that standard M-H has probability zero of sampling a conformation that satisfies terminal endpoint constraints exactly, and is not applicable to closed loops. So, these tests ignore the loop closure constraint altogether.

**Figure 17:**
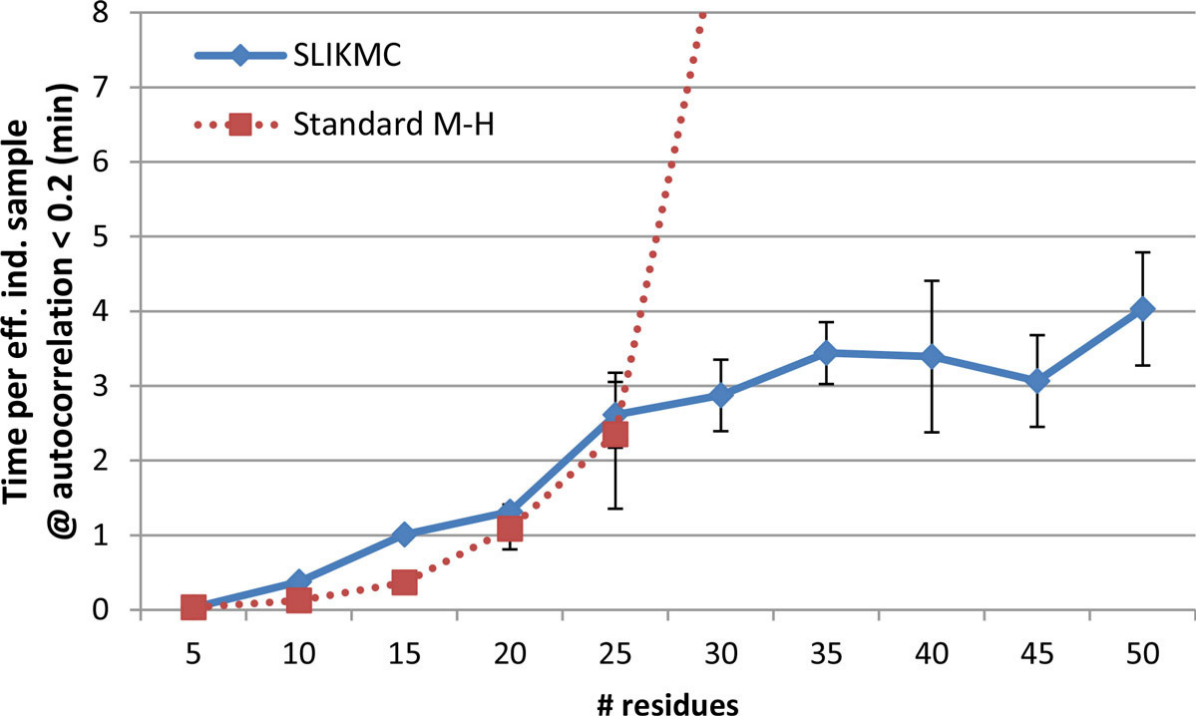
**Running time comparison between SLIKMC and standard Metropolis-Hastings**. Time required to obtain one quasi-independent sample on open-ended subchains of 1B8C with a variety of lengths. Standard M-H did not generate even one sample for chain lengths above 30 after 30 minutes.

Figure 17 displays the average time needed to obtain one quasi-independent sample over ten 30-minute runs for different chain lengths. The skip lengths are determined empirically for each run. This data suggests that our method achieves a cost per quasi-independent sample that is nearly linear to the length of the chain. In contrast, the likelihood that standard M-H accepts a sample drops dramatically as the number of residues increases, leading to exponentially growing cost per sample.

### Simultaneous backbone and side-chain sampling

**Figure 18:**
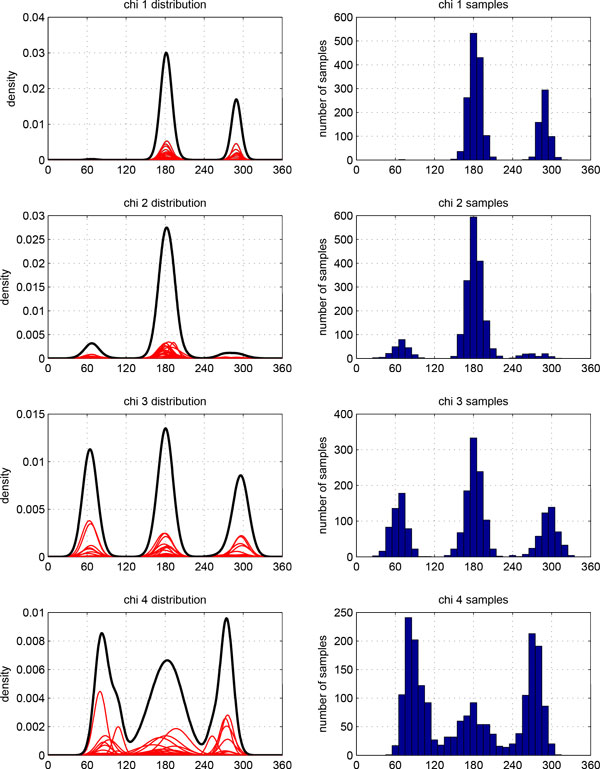
**Side-chain distribution of residue ARG**. Left: Gaussian mixture distribution of side-chain torsion angles for the native structure of residue 130 (arginine) in protein 1AMP. Right: histograms of side-chain angles from samples generated by SLIKMC. The distributions of *χ*_1_, *χ*_2 _match well with the sampling distributions while the distributions of *χ*_3 _and *χ*_4 _are limited due to steric clashes.

We demonstrate backbone and side-chain sampling using a 15-residue helix structure 1AMP 120-134. As priors we use backbone-dependent rotamer distributions, Ramachandran plot priors, B-factors for the backbone, and testing self-collision and collision against the non-loop portion of the chain. Given 20 min cutoff time, 1,623 samples are generated. Figure 18 illustrates that in residue 130 (arginine), the distributions of torsional angles *χ*_3 _and *χ*_4 _are limited due to steric clashes, while *χ*_1 _and *χ*_2 _match well with the priors.

## Conclusion

We propose SLIKMC - a Markov chain Monte Carlo method for sampling closed chains according to specified probability distribution. A probabilistic graphical model (PGM) is proposed to specify the structure preferences. A novel method for sampling sub-loops is developed to generate statistically unbiased samples of probability densities restricted by loop-closure constraints and mathematical conditions necessary for unbiased sampling is derived. Simulation experiments show that SLIKMC completes large loops (*>*10 residues) orders of magnitude faster than standard Monte Carlo and discrete search techniques.

SLIKMC is demonstrated to be applicable to various tasks such as conformation ensemble generation, missing structure construction. For future work we intend to integrate SLIKMC with more complex energy functions, statistical potentials, and machine-learning-based structural function predictors. Another limitation of the technique is that due to the locality of each block adjustment, large-magnitude global motions may take a huge number of iterations to sample, particularly when the motion must cross low-scoring chasms in conformation space. We intend to investigate annealing-like or random restart techniques for overcoming these difficulties, as well as different block choices that allow the algorithm to take larger steps. Finally, we are interested in extending our method to study simultaneous backbone and side-chain flexibility in protein-ligand and protein-protein binding.

## Appendix

This appendix presents a fundamental statement about probability densities under a transformation of variables.

*Suppose *$u\in {\mathbb{R}}^{m}$*and *$v\in {\mathbb{R}}^{n}$*are multivariate random variables related by ***v **= *f *(**u**), *where f is differentiable and injective. Denote the image of *$A\subseteq {\mathbb{R}}^{m}$*as *$M=f\left(A\right)\subseteq {\mathbb{R}}^{m}$. *If g_u _is a density with support over A, then the corresponding density over M, with respect to the m-volume measure, is*

(19) $$g_{v}\left(v\right)=g_{u}\left(f^{-1}\left(v\right)\right)/\sqrt{\text{det}G\left(f^{-1}\left(v\right)\right)}$$

*where G*(**u**) *is the metric tensor:*

(20) $$G\left(u\right)={\left(\frac{\partial f}{\partial u}\left(u\right)\right)}^{T}\left(\frac{\partial f}{\partial u}\left(u\right)\right).$$

More precisely, g_v _as defined above satisfies:

(21) $$\int_{f\left(U\right)}g_{v}\left(v\right)d\mu =\int_{U}g_{u}\left(u\right)du$$

*for any subset U *⊆ *A, where dµ is the m-volume element of M*.

From change of variables we have:

(22) $$\int_{f\left(U\right)}g_{v}\left(v\right)d\mu =\int_{U}g_{v}\left(f\left(u\right)\right)X\left(u\right)du$$

where *X*(**u**) is the *m*-volume of the parallelotope spanned by the axes of the coordinate chart *f *centered at **u**: $\frac{\partial f}{\partial u_{1}}\left(u\right),...\frac{\partial f}{\partial u_{m}}\left(u\right)$.

We now use the fact that the *m*-volume *V *of the parallelotope spanned by *m *vectors $v_{\text{1}},...,v_{m}\in \mathbb{R}$ is given by the determinant:

(23) $$V^{2}=\text{det}\left(\begin{array}{cccc} v_{1}^{T}v_{1} & v_{1}^{T}v_{2} & \cdots \; & v_{1}^{T}v_{m}\\ v_{2}^{T}v_{1} & v_{2}^{T}v_{2} & \cdots \; & v_{2}^{T}v_{m}\\ \vdots & & \vdots & \\ v_{m}^{T}v_{1} & v_{m}^{T}v_{2} & \cdots \; & v_{m}^{T}v_{m} \end{array}\right).$$

Note that this can be expressed more compactly as det(*A^T ^A*) where *A *is the matrix with **v**_1_, ..., **v***_m _*as its columns. Hence, $X\left(u\right)=\sqrt{\text{det}G\left(u\right)}$. Finally, substituting *g_u _*in the r.h.s. of (22) gives the desired result.

## Competing interests

The authors declare that they have no competing interests.

## Authors' contributions

YZ implemented the algorithm and conducted the numerical experiments. KH contributed to the study design and developed the mathematical foundations. All authors contributed to drafting the manuscript and approved the final manuscript.

## Declarations

The publication costs for this article were funded by Dr. Kris Hauser.

This article has been published as part of *BMC Structural Biology *Volume 13 Supplement 1, 2013: Selected articles from the Computational Structural Bioinformatics Workshop 2012. The full contents of the supplement are available online at http://www.biomedcentral.com/bmcstructbiol/supplements/13/S1.
